# Supplementary material for: Parental and peer support and modelling in relation to domain-specific physical activity participation in boys and girls from Germany
Source: PLoS One. 2019 Oct 30;14(10):e0223928. doi: 10.1371/journal.pone.0223928 (PMC6821055; doi:10.1371/journal.pone.0223928)
Supplement: Attachment — Submitted filename: SocSupport response to review 11 09 2019.docx [file pone.0223928.s001.docx]

**Response to review: Ms. No. PONE-D-19-17332**

**PLOS ONE**

Dear Colleagues,

Please find enclosed our revision of the following research article:

“Parental and peer support and modelling in relation to domain-specific physical activity participation in boys and girls from Germany”

We would like to thank the reviewers for their comments. We believe that the quality of our manuscript has improved by implementing the suggestions and comments received. We hope we have addressed all the reviewers’ concerns and comments to your satisfaction.

Please find our response to the comments below. The reviewers’ comments are shown in regular font, and our responses are shown below each comment in *italic*:

**Reviewer #1:**

Introduction:

1. Really nice integration of theory in the background. Overall nice review of literature.

*Thank you.*

2. Page 3, line 43 - adiposity is not a disease, it is a risk factor

*We deleted adiposity from the list of diseases.*

3. Page 4, line 66 - change "probably" to "likely" or another word

*Done.*

4. Page 4, line 67 - change "typically" to "typical"

*Done.*

5. Page 5, line 95 - change "form" to "from"

*Done.*

6. Page 5, line 96 - spell out MVPA first time

*Done.*

7. Page 6, lines 119-120 - what is meant by different domains and domain-specific? This is explained in the methods, but should be introduced in this section as well

*We added some more information and examples.*

Material and Methods

8. Page 7, line 147 - remove comma after children

*Done.*

9. Page 8, line 170-171 - say this in the intro as examples of domains (same as comment above)

*See above.*

10. Page 8-9 - for PA in sports clubs, PA outside sports clubs, and extra-curricular PA, why were these variables dichotomized rather than used as continuous? The cutoff of 0 vs 1 or more minutes seems very arbitrary. Is this how these variables have been used in the past? I would suggest using these variables as continuous and linear rather than logistic regression for these three outcomes.

*We agree with you and think, it could be also interesting to investigate the relationship of social environmental factors with the amount (level) of physical activity. However, it is also possible to consider physical activity participation as a dichotomous variable (**1**) and we decided to focus on whether or not the children and adolescents participated in physical activity in different domains due to various reasons: we focused on a large number of different domains of physical activity of which some are commonly measured in a dichotomous way (active vs. non-active; participation vs. non-participation). For example, in active transportation research it is very common to investigate whether a person is actively commuting or not (**2-4**) and research on correlates/determinants of active transportation focuses on the question which factors determine whether a person is an active or passive commuter. Regarding extra-curricular physical activity there is a similar situation (**5-7**). However, previous research on overall sports or physical activity participation also used dichotomised PA items (**8-11**). In addition, on a theoretical basis this is based on the assumption that there are different correlates/determinants of sports/physical activity participation (active vs. non-active) and of the amount of daily/weekly exercise/physical activity. Our paper focusses on participation and we revised the manuscript to make this even more conspicuous throughout the manuscript.*

11. Page 14 - "...girls had a lower prevalence in physical activity..." - prevalence is not the right word here

*We revised this sentence.*

12. Page 15 - Table 2 - Revise title - "Social support, social modeling, and domain-specific physical activity by gender and grade level"

*Done.*

13. Page 15 - Table 2 - Peer support, parental support, and peer modeling - doesn't make sense to me to show these as means/SD, I think you should show percents in each category as these are categorical variables.

*Our social support scales are based on more than one item (2 or 3) and thus, contain calculated mean values. That is why we presented the mean/SD in our tables as also done in other studies before (**12-14**).*

14. PA in sports clubs, PA outside sports clubs, and extra-curricular PA should be shown as mean number of minutes rather than "% yes". For outdoor play, if it is going to be shown as dichotomous, specify that this is the percent who said 4+ days/week.

*We revised the column with the information on the units given in the table. See also our answer to your comment no. 10.*

15. Tables 3-5 - should be multivariate linear regression

*See our answer to your comment no. 10.*

Discussion, Strengths/limitations, conclusion

16. First paragraph, first sentence - split into more than one, it is long right now

*Done.*

17. Second paragraph, first sentence - revise, it is unclear

*We revised the sentence.*

18. Page 28, last sentence - should it say shift rather than swift?

*We corrected this typo.*

19. Page 30, last sentence - Corti, not Gorti

*We corrected this typo.*

20. Add more implications for intervention work in the discussion and/or conclusion, or even a separate section

*We added some implications for interventions in the discussion and conclusion.*

**Reviewer #2:**

General Comment:

21. This is an interesting study conducted with a large number of adolescents from Germany.

*Thank you.*

22. However some aspects can still be improved in the manuscript and there are some doubts that can be better clarified throughout the text.

*Thank for your comments. We hope that, in the revised manuscript, we have addressed all your concerns and comments to your satisfaction.*

Further comments:

23. In the results section in the abstract, enter more information with the continuous values of the main results.

*We added some more information on the estimates of our analyses in the abstract.*

24. The introduction is too long, some paragraphs could be reduced to make reading more dynamic.

*We revised and shortened the introduction. However, by having the first comment from the first reviewer in mind we retained our review of the literature.*

25. In the introduction, it needs to be made clear where this study is progressing compared to articles on this topic previously published in the literature.

*We revised the penultimate and last paragraph of the introduction to embed our study into the state of research.*

26. Please insert in the methods the sample size calculation used for this study.

*The MoMo Wave 1 Study is part of the longitudinal German Health Interview and Examination Survey for Children and Adolescents, KiGGS. The survey aims to investigate and monitor health and health behaviour in children and adolescents from Germany and encompasses over 17,000 and 12,000 children and adolescents in the baseline and wave 1 data collection, respectively. In the MoMo Study as a module study of KiGGS a large proportion of the KiGGS participants could participate (in wave 1: nearly 4,000). For our research question we analysed the data from the MoMo participants that was available. Thus, no specific sample size calculation has been conducted for the research question at hand.*

27. “Extra-curricular physical activity was assessed by a question about whether the participants attend in extra-curricular physical activities with frequency, type of activity and duration. A dichotomous variable “extra-curricular physical activity” was built according to 1–“one or more minutes extra-curricular physical activities” or 0–“no extra-curricular physical activities”. What types of extracurricular physical activities would be considered? Please explain this further in the methods section.

*We added some more information.*

28. A question in the methods section, did only the teens answer the questionnaires or did the teens' parents also answer some questions about social support for their children's physical activity? If so, this needs to be better addressed in the methods section.

*No, the teens answered the questionnaires without the parents as stated in the methods section (Data collection). Just in young children up to the age of 11 had the help of their parents (but the parents did not answer some questions without the children). For a better understanding, we improved the wording in this paragraph.*

29. “Participants with separated parents were assigned the socioeconomic status of the parent they lived with. All three aspects income, educational and professional status were scored on a scale from 1 to 7 and a sum score was created (range: 3–21) and categorized into low (3–8), medium (9–14) and high (15–21) socioeconomic status [57].” This is an important aspect in the present study, please, would like the authors to present the analysis considering children of parents living together and in the other analysis children of separated parents. My question is whether children of parents living together could be more physically active when bought from children of separated parents?

*We agree with you that this could be a very interesting and relevant question. However, we do not have in depth data on the family model and the main caregiver. Thus, we included this relevant as an implication in the discussion section.*

30. In the results section, would the authors have social support information regarding parental gender? If so, it would be pertinent to analyze this relationship stratified: example: fathers x daughters; fathers x son; mothers x son; mothers x daughters.

*Unfortunately, we do not have data on parental support for mothers and fathers separately.*

31. “These findings were expected, given the fact that previous studies also presented gender differences in overall physical activity or moderate to vigorous physical activity that became more apparent in the transition from childhood to adolescence [6,9,58,59].” Why would this happen? The authors need to advance in this aspect in the discussion.

*We revised this paragraph and gave some deeper insights into this topic.*

32. “Another possibility is, that higher levels of physical activity in boys which have been found in many studies [5,6,9], require higher levels of social support, which could lead to a higher readiness of parents or peers to provide support [60]. Therefore, it is possible that boys receive more parental support for physical activity than girls because they claim for more support to conduct their activities”. Would not the practice of previous physical activity of parents in their own childhood and adolescence be a factor to be considered in this relationship? Would the more physically active father throughout his life have a greater chance of his son being more physically active? So would the relationship between mothers and daughters? There is information in this sense in the literature that could be inserted as an important aspect in the discussion.

*This is a very relevant and interesting point. We agree with you that fathers are more physically active than mothers (as stated in our manuscript in the following paragraph). However, in our study girls reported similar (in case of paternal modelling rated by secondary school-children) or higher levels of maternal and paternal modelling than boys (see table 2). Boys did not perceive to have more active fathers than girls did and additionally, boys did not perceive to have more active same-sex models than girls.*

*However, we revised the paragraph to sufficiently deal with this important point.*

33. What are the practical applications of this study?

*See our answer to comment no. 20 of reviewer #1.*

1.	Welk GJ. Physical activity assessments for health-related research. Champaign, Ill.: Human Kinetics; 2002.

2.	Pabayo R, Gauvin L, Barnett TA. Longitudinal Changes in Active Transportation to School in Canadian Youth Aged 6 Through 16 Years. Pediatrics. 2011;128(2):E404-E13.

3.	Titze S, Giles-Corti B, Knuiman MW, Pikora TJ, Timperio A, Bull FC, et al. Associations Between Intrapersonal and Neighborhood Environmental Characteristics and Cycling for Transport and Recreation in Adults: Baseline Results From the RESIDE Study. J Phys Act Health. 2010;7(4):423-31.

4.	Bere E, van der Horst K, Oenema A, Prins R, Brug J. Socio-demographic factors as correlates of active commuting to school in Rotterdam, the Netherlands. Prev Med. 2008;47(4):412-6.

5.	Ara I, Vicente-Rodriguez G, Perez-Gomez J, Jimenez-Ramirez J, Serrano-Sanchez JA, Dorado C, et al. Influence of extracurricular sport activities on body composition and physical fitness in boys: a 3-year longitudinal study. Int J Obes. 2006;30(7):1062-71.

6.	Gracia-Marco L, Tomas C, Vicente-Rodriguez G, Jimenez-Pavon D, Rey-Lopez JP, Ortega FB, et al. Extra-curricular participation in sports and socio-demographic factors in Spanish adolescents: the AVENA study. J Sports Sci. 2010;28(13):1383-9.

7.	La Torre G, Masala D, De Vito E, Langiano E, Capelli G, Ricciardi W, et al. Extra-curricular physical activity and socioeconomic status in Italian adolescents. BMC Public Health. 2006;6:9.

8.	Mader U, Martin BW, Schutz Y, Marti B. Validity of four short physical activity questionnaires in middle-aged persons. Med Sci Sports Exerc. 2006;38(7):1255-66.

9.	Mota J, Almeida M, Santos P, Ribeiro JC. Perceived Neighborhood Environments and physical activity in adolescents. Prev Med. 2005;41(5-6):834-6.

10.	Manz K, Mensink GBM, Jordan S, Schienkiewitz A, Krug S, Finger JD. Predictors of physical activity among older adults in Germany: a nationwide cohort study. BMJ Open. 2018;8(5):e021940.

11.	Best K, Ball K, Zarnowiecki D, Stanley R, Dollman J. In Search of Consistent Predictors of Children's Physical Activity. Int J Environ Res Public Health. 2017;14(10).

12.	Mutz M, Albrecht P. Parents' Social Status and Children's Daily Physical Activity: The Role of Familial Socialization and Support. Journal of Child and Family Studies. 2017;26(11):3026-35.

13.	Eime RM, Casey MM, Harvey JT, Sawyer NA, Symons CM, Payne WR. Socioecological factors potentially associated with participation in physical activity and sport: A longitudinal study of adolescent girls. J Sci Med Sport. 2015;18(6):684-90.

14.	Trost SG, Sallis JF, Pate RR, Freedson PS, Taylor WC, Dowda M. Evaluating a model of parental influence on youth physical activity. Am J Prev Med. 2003;25(4):277-82.
